# Quantity over quality—Findings from a systematic review and environmental scan of patient decision aids on early abortion methods

**DOI:** 10.1111/hex.12617

**Published:** 2017-09-07

**Authors:** Kyla Z. Donnelly, Glyn Elwyn, Rachel Thompson

**Affiliations:** ^1^ The Dartmouth Institute for Health Policy and Clinical Practice Dartmouth College Lebanon NH USA

**Keywords:** abortion, decision aid, environmental scan, informed choice, pregnancy termination, systematic review

## Abstract

**Background:**

The availability and effectiveness of decision aids (DAs) on early abortion methods remain unknown, despite their potential for supporting women's decision making.

**Objective:**

To describe the availability, impact and quality of DAs on surgical and medical early abortion methods for women seeking induced abortion.

**Search strategy:**

For the systematic review, we searched MEDLINE, Cochrane Library, CINAHL, EMBASE and PsycINFO. For the environmental scan, we searched Google and App Stores and consulted key informants.

**Inclusion criteria:**

For the systematic review, we included studies evaluating an early abortion method DA (any format and language) vs a comparison group on women's decision making. DAs must have met the Stacey et al (2014). Cochrane review definition of DAs. For the environmental scan, we included English DAs developed for the US context.

**Data extraction and synthesis:**

We extracted study and DA characteristics, assessed study quality using the Effective Practice and Organization of Care risk of bias tool and assessed DA quality using International Patient Decision Aid Standards (IPDAS).

**Results:**

The systematic review identified one study, which found that the DA group had higher knowledge and felt more informed. The evaluated DA met few IPDAS criteria. In contrast, the environmental scan identified 49 DAs created by non‐specialists. On average, these met 28% of IPDAS criteria for Content, 22% for Development and 0% for Effectiveness.

**Conclusions:**

Research evaluating DAs on early abortion methods is lacking, and although many tools are accessible, they demonstrate suboptimal quality. Efforts to revise existing or develop new DAs, support patients to identify high‐quality DAs and facilitate non‐specialist developers' adoption of best practices for DA development are needed.

## 1 INTRODUCTION

As we have described previously,^1^ women in the United States value receiving quality information^2^, ^3^, ^4^ and support^5^ when making decisions about early abortion.^6^, ^7^, ^8^, ^9^ The two recommended methods, surgical and medical abortion, are both highly effective, safe and acceptable, yet differ across several aspects (eg duration, bleeding and cramping profile, where the abortion takes place, follow‐up visit requirements).^10^ According to a recent national survey of abortion providers,^11^ both methods are available in a majority of abortion facilities, and among those facilities that offer only the medical option, most are located in areas also served by those that offer surgical abortion.^11^ Recent changes to the US Food and Drug Administration regulations for mifepristone‐misoprostol medical abortion are also anticipated to expand access to women.^12^ Given the potential availability of both methods, and that they are similarly effective yet encompass very different processes, women's preferences and circumstances are paramount to their method choice.

Decision aids are tools designed to support patients to compare medically appropriate options and make informed decisions based on their preferences and quality evidence.^13^ They enable standardized, patient‐centred and balanced information provision and have been shown to improve patients' knowledge, participation in decision making and the alignment between their choices and their values in a range of clinical settings.^13^ Decision aids are particularly relevant for the early abortion context given the prevalence of biased information about abortion,^14^ relatively poor knowledge about the safety and consequences of abortion among the general public^15^, ^16^ and the diminishing access to qualified abortion providers^17^ who often serve as an important source of trusted information.^16^ Moreover, because primary care providers sometimes have insufficient knowledge^18^ and training^19^ in early abortion methods, and counselling is not always well received from abortion providers,^20^ integrating a consistent, high‐quality decision aid may help health professionals across disciplines to better support women's decision making process. Even in areas where there may not be ready access to both methods, a decision aid may enable women to develop accurate expectations about the method that will be used and may also empower women as consumer advocates. Despite the potential utility of a decision aid on early abortion methods, we lack knowledge on the availability, quality and impact of existing decision aids on this topic.

The most recent Cochrane review of decision aids^13^ and two other systematic reviews^21^, ^22^ identified a single study evaluating an early abortion method decision aid,^23^ which is no longer available to the public. However, none of these reviews assessed the quality of identified decision aids. Additionally, because these reviews included only randomized controlled trials, whether other decision aids have been developed and evaluated using non‐randomized study designs or developed and evaluated but not published in the scientific literature is unknown. What also remains unclear is the availability and quality of decision aids that have not undergone evaluation, including those developed by entities without specialist expertise in decision aid design (eg abortion clinics). Such decision aids may be more easily accessed by the general public and thus more likely to be the first‐line source of information.

The two objectives of this study were (i) to conduct a systematic review to identify, appraise and evaluate the impact of early abortion method decision aids described in the scientific literature, and (ii) to conduct an environmental scan of the grey literature to identify and appraise other early abortion method decision aids developed in the United States.

## 2 METHODS

Study methods are described in detail in a published study protocol^1^ and summarized below.

### 2.1 Systematic review (Part I)

We registered the systematic review protocol on 12 February 2015 with the International Prospective Register of Systematic Reviews (CRD42015016717), and this reporting adheres to the PRISMA methodology.^24^

#### 2.1.1 Inclusion and exclusion criteria

We included studies if they were randomized controlled trials or non‐randomized, cohort, case‐control, before‐and‐after, interrupted time series or repeated‐measures studies.^25^ They must have included women eligible for and facing a decision between medical and surgical abortion (as defined by trialists), and collected patient‐ or observer‐reported data on the impact of an early abortion method decision aid on women's decision making processes or outcomes. Our primary outcome was decision quality, defined as the extent to which a patient's decision is informed and based on personal values.^26^, ^27^, ^28^

The decision aid must have met the definition adopted in the Cochrane review of decision aids available at the time the study was designed (ie “interventions designed to help people make specific and deliberative choices among options (including the status quo), by making the decision explicit and by providing (at the minimum) (i) information on the options and outcomes relevant to a person's health status, and (ii) implicit methods to clarify values” (Stacey et al, p. 8^29^)), compared medical and surgical early abortion methods,^1^ been publicly available (ie free) and been developed after 2000, when medical abortion became legal in the United States.^30^ The decision aid could have been designed for use at any time, in any format (eg electronic documents, static websites, interactive websites, videos, DVDs, pamphlets, booklets, smartphone mobile applications (“apps”)) and in any language.

After finalization of the study protocol,^1^ we elected also to exclude studies of women whose gestational age was unclear and studies evaluating decision coaching not accompanied by a physical tool.^31^

#### 2.1.2 Search strategy

We searched MEDLINE/PubMed, The Cochrane Library, CINAHL, EMBASE and PsycINFO using, where appropriate, medical subject heading (MeSH) terms: “abortion, induced,” “patient education,” “choice behavior,” “decision making” and “decision support techniques” and/or key words with Boolean operators. We did not apply any language limits but, reflecting decision aid inclusion criteria, we searched only for studies published since January 2000, as described above. We also searched the trial registry ClinicalTrials.gov, manually searched the reference list of the included article (seeResults) and considered for inclusion any decision aid identified from the environmental scan that had been evaluated and published in a peer‐reviewed outlet. We also reviewed any articles identified in Google Scholar as having cited the included article. All searches were conducted in February 2015 (search results included in AppendixS2).

#### 2.1.3 Screening process

The screening and full review process of Internet pages and apps is described in full in the protocol.^1^ We had planned to screen both titles and abstracts of all articles after duplicate entries were removed,^32^ but ultimately opted to screen only titles unless further clarification from the abstract was needed. This screening approach has been shown to be as precise and more efficient than screening both titles and abstracts.^33^ The primary reviewer (KD) classified each article as “potentially eligible” or “ineligible” for inclusion and, for articles classified as “ineligible,” recorded the most salient reason. We had planned that the secondary reviewer (RT) would independently screen random samples of 10% of the titles and/or abstracts in each classification (ie “potentially eligible” and “ineligible”). However, because the primary reviewer only identified 12 “potentially eligible” studies (seeResults ), the second reviewer independently screened all of these studies (in addition to the 10% of “ineligibles”). The reviewers' classifications matched exactly. The same process was used for full‐text review.

#### 2.1.4 Data extraction

The primary and secondary reviewers used a customized form to independently extract data on the study design, participant characteristics, decision aid characteristics (eg format, mode of administration) and outcomes for the identified study. Abstracted data were compared and disagreements resolved by discussion.

#### 2.1.5 Study and evidence quality appraisal

Both reviewers independently assessed the methodological quality using the Cochrane Effective Practice and Organization of Care Group's (EPOC) risk of bias criteria.^34^

#### 2.1.6 Decision aid quality appraisal

The quality of the decision aid evaluated in the included study was assessed by the primary and secondary reviewers using the 2005 version of the International Patient Decision Aid Standards (IPDAS) checklist.^35^ This checklist includes items in the Content domain (ie the information, probabilities, values clarification and guidance in deliberation specific to the health condition), Development domain (ie the design and development process) and Effectiveness domain (ie outcomes related to a high‐quality decision).^35^ Because the included study did not comprise decision aids on a diagnostic test, there were 59 potentially relevant items, including supplementary items for Internet‐based tools (6 items) and tools that included patient stories (3 items). On the quality appraisal form developed for this study, minor clarifications or examples were added to some checklist items to improve clarity and thus consistency in appraisal across reviewers (form available in AppendixS1). Items that could not be confidently assessed with the information available were coded as not having met the criteria. Items that were not applicable were coded as such.^2^

We used the Flesch‐Kincaid test analytics in Microsoft Word to assess the readability and reading ease (scale 0‐100, higher is easier to read) of text‐based decision aids.^36^, ^37^ We chose a readability level of 8th grade or below to indicate limited reading skills IPDAS (Item 42) and to correspond with the mean reading level in the US population.^38^

#### 2.1.7 Analysis

We had planned to perform a meta‐analysis of study results, but only one eligible study was identified.

### 2.2 Environmental scan (Part II)

#### 2.2.1 Inclusion and exclusion criteria

For the environmental scan, we adopted all intervention inclusion and exclusion criteria used in the systematic review and imposed two further criteria, excluding decision aids not written in English and those not created by a source in the United States, for women living in the United States.

#### 2.2.2 Search strategy

We conducted four Google searches using the following search strings: (i) *abortion options,* (ii) *abortion decision aid*, (iii) *medical or surgical abortion*, and (iv) *pregnancy termination options*. We searched the Apple App Store and Google Play^39^ using the key word *abortion*. We also solicited information about decision aids via the National Abortion Federation and Abortion Care Network listservs and Twitter, emailed key informants who work in abortion care and/or research (ie The American Congress of Obstetricians and Gynecologists, Planned Parenthood Federation of America, Reproductive Health Access Project), and reviewed the Ottawa Hospital Research Institute's Decision Aid Library Inventory.^40^ Additionally, the decision aid identified during the systematic review was considered for the environmental scan. All searches were conducted in February 2015.

#### 2.2.3 Screening process

The screening and full review process of Internet pages and apps is described in full in the protocol.^1^ We had planned to classify tools as “eligible” or “ineligible” for inclusion, but elected to add “unclear” as a third classification due to unforeseen challenges in categorizing some of the resultant tools. We contacted two study authors for clarifying information about the study population, outcomes and intervention.

The inter‐rater reliability of eligible and ineligible classifications between the primary and secondary was calculated to be κ = 0.74, and thus exceeded our minimum requirement (0.7). For those classified as “unclear,” the primary and secondary reviewers came to a decision about eligibility together.

#### 2.2.4 Data extraction

The primary reviewer used a customized form to extract data from all eligible decision aids (eg format, characteristics and source).

#### 2.2.5 Decision aid quality appraisal

The primary reviewer appraised the quality of all included decisions using the IPDAS checklist and the Flesch‐Kincaid tests. The secondary and tertiary (GE) reviewers independently appraised the quality of random samples of 10% of the eligible decision aids. Inter‐rater reliability for decision aid quality appraisals was calculated to be κ = 0.74 and κ = 0.85, and again exceeded our minimum requirement.

## 3 RESULTS

### 3.1 Systematic review

**Figure 1:**
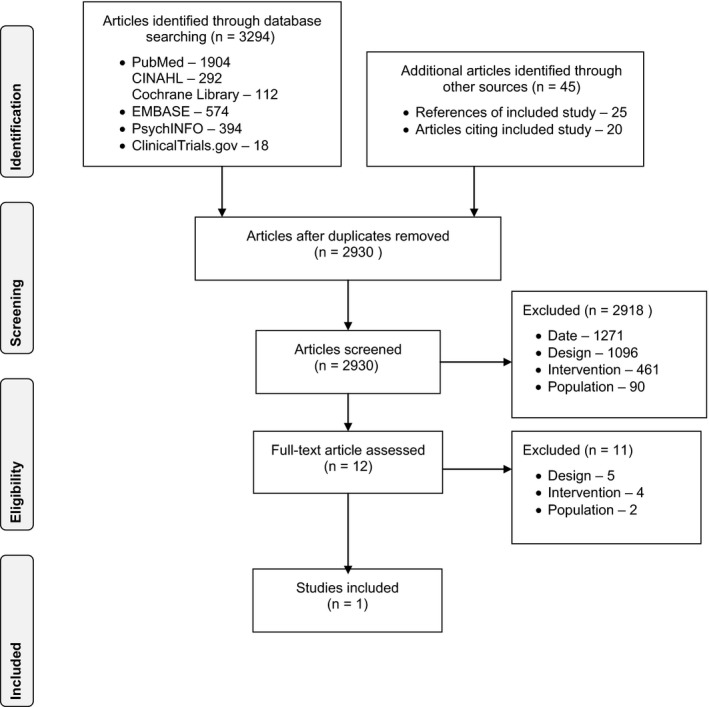
Systematic review PRISMA flow diagram

Altogether, 2930 unique articles were identified through database searches and other search methods. Of those articles classified as “potentially eligible” for inclusion, only one described a study that met all eligibility criteria (see Figure 1). This study was the randomized controlled trial identified in prior systematic reviews.^13^, ^21^, ^22^ It was conducted in 2002 in the United Kingdom and randomized women to receive either a three‐page paper decision aid about early abortion methods (n = 163) or a control leaflet about contraception (n = 165) in the waiting room before an abortion counselling consultation. We contacted the corresponding author to clarify the study outcomes and to inquire about related publications (eg study protocols, companion studies), none of which were identified.

#### 3.1.1 Decision aid impact

The included study found that women randomized to receive the decision aid had higher levels of knowledge about both methods, had more favourable scores on the Informed subscale of the Decisional Conflict Scale and expressed more positive attitudes towards medical abortion than women randomized to receive the control leaflet. No differences were found between groups in scores on the Uncertain or Effectiveness subscales of the Decisional Conflict Scale, level of anxiety, attitudes towards surgical abortion or chosen abortion method. Although there were mixed findings about the effect of group on risk perception scores about each method, generally, women in the decision aid group had lower scores. Other study outcomes are described in the Cochrane Collaboration review of decision aids^13^ and the two other systematic reviews mentioned previously.^21^, ^22^

#### 3.1.2 Decision aid quality appraisal

The decision aid met 5 of 23 IPDAS criteria for the Content domain of the IPDAS checklist, 11 of 20 for Development and 1 of 7 for Effectiveness. The Flesch‐Kincaid readability was calculated to be US grade level 6.9 and 62.9 reading ease.

#### 3.1.3 Study and evidence quality appraisal

Using the EPOC risk of bias criteria, the study was classified as having low risk of bias in five of nine domains (see Table 1). The study was classified as having high risk of bias in two domains: contamination (because randomization was at the patient level) and selective outcome reporting (because they used the entire Decisional Conflict Scale but only reported select subscales). The risk of bias was unclear for similar baseline characteristics (because no baseline outcome measurements were obtained) and for blinding (because no primary outcome was specified). No other sources of potential bias were identified.

**Table 1:** Effective Practice and Organization of Care risk of bias assessment

Criteria	Unclear	Low	High
Sequence generation		✓	
Allocation concealment		✓	
Similar baseline outcome measurements	✓		
Similar baseline characteristics		✓	
Blinding	✓		
Incomplete outcome data		✓	
Contamination			✓
Selective outcome reporting			✓
Other sources of potential bias		✓	

Due to the identification of only one study, we were not able to use the Grading of Recommendations Assessment, Development and Evaluation (GRADE) criteria to rate the quality of evidence as planned.^41^

### 3.2 Environmental scan

**Figure 2:**
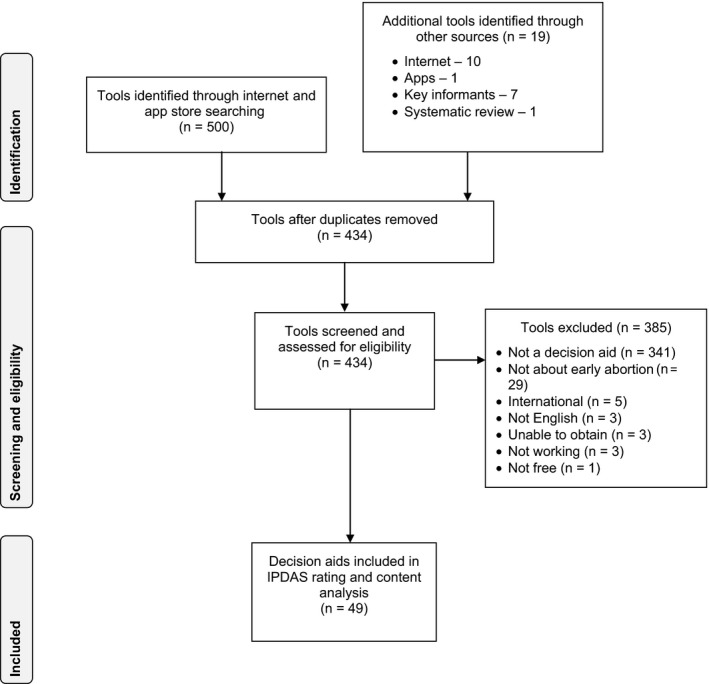
Environmental scan PRISMA flow diagram

Altogether, 434 unique tools were identified through Internet and App Store searches and other sources. Of these, 49 met eligibility criteria (see Figure 2). Three sources referenced a decision aid, but we were unable to obtain them despite attempts to contact the authors. The vast majority of the excluded tools did not meet the definition of a decision aid (ie they failed to make the decision explicit and/or to include implicit methods to clarify values (n = 341)).^29^ The decision aid identified in the systematic review did not meet eligibility criteria for the environmental scan because it was not created for women in the United States.

#### 3.2.1 Decision aid characteristics

Most of the included decision aids (n = 42) were non‐interactive multipage websites that averaged 14.6 pages in length (range 2‐208 pages). The remaining decision aids comprised 4 Apple Smartphone apps, 2 PDFs and 1 video. All decision aids appeared to be created by entities without specialist expertise in decision aid development, including 32 by abortion services, seven by reproductive health‐related organizations, four by consumer health information organizations, four by pregnancy clinics that do not provide abortion procedures or referral, one by a clinician and one by a patient advocate. All decision aids compared at least one method of early surgical and medical abortion, with the majority describing some type of early surgical abortion to medical abortion with mifepristone and misoprostol. Three decision aids also compared manual vacuum aspiration to electrical vacuum aspiration. Thirteen decision aids also described methotrexate and one, tamoxifen, as alternatives to mifepristone.

#### 3.2.2 Decision aid quality

On average, the decision aids met the criteria for 28% (n = 6) of the 23 items for Content (range: 3‐12) and 0% (n = 0) of the 7 items for Effectiveness domains (see Figure 1). Due to the nature of the included decision aids, the number of applicable items in the Development domain varied from 18 to 27, with an average of 22% (n = 5) of items met (range: 2‐11). Table 2 provides an overview of the average scores (and ranges) for different types of decision aids. A table of scores by each decision aid is available upon request.

**Table 2:** Average International Patient Decision Aid Standards scores for different types of decision aids

Decision aid type	Content	Development	Effectiveness
Website with narratives (n = 4)	6.5/23 = 28% (range: 6‐9)	6.5/27^a^ = 24% (range: 6‐7)	0/7 = 0%
Website without narratives (n = 38)	6.6/23 = 29% (range: 3‐12)	5.6/24^b^ = 23% (range: 2‐11)	0/7 = 0%
PDF (n = 2)	6/23 = 26% (range: 6)	4.5/20^c^ = 23% (range: 4‐5)	0/7 = 0%
Smartphone app (n = 4)	4/23 = 17% (range: 4)	4/23^d^ ** **= 17% (range: 4)	0/7 = 0%
Audiovisual (n = 1)	7/23 = 30%	2/18^e^ ** **= 11%	0/7 = 0%

The denominator varied based on the number of applicable IPDAS items. Items that were not applicable are indicated as such: ^a^46, 47; ^b^46, 47, 50, 51, 52; ^c^44, 45, 46, 47, 48, 49, 50, 51, 52; ^d^46, 47, 48, 50, 51, 52; ^e^41, 42, 44, 45, 46, 47, 48, 49, 50, 51, 52.

The decision aids met more IPDAS items in the Content and Development domains than in the Effectiveness domain, as described in Table 3 with clarifying comments.

**Table 3:** Number of decision aids that met each International Patient Decision Aid Standards (IPDAS) criteria

IPDAS criteria	n	Comments
*Content*		
Describe the health condition	32/49	Sometimes failed to explain that abortion is performed to end a pregnancy.
List the options	49/49	
List the option of doing nothing	18/49	Majority did not list the alternative options (ie adoption or continuing the pregnancy).
Describe the natural course without options	6/49	Few included information about the process of continuing the pregnancy.
Describe procedures	38/49	
Describe positive features	35/49	
Describe negative features of options	46/49	
Include chances that positive and negative outcomes may happen	0/49	Descriptions typically comprised qualitative information (eg “you may experience heavy bleeding”) instead of probabilities on the likelihood of experiencing certain outcome.
Use event rates specifying the population and time period	0/49	Quantitative data were used selectively (eg success or failure rates were commonly provided, but not rates for other positive and negative outcomes).
Compare outcome probabilities using the same denominator, time period, scale	0/49	
Describe uncertainty around probabilities	18/49	Descriptions were usually qualitative (eg “you cannot predict what exactly will happen to you” or “the [side effect] may happen”).
Use visual diagrams	1/49	
Use multiple methods to view probabilities (words, numbers, diagrams)	0/49	
Allow the patient to select a way of viewing probabilities	0/49	
Allow the patient to view probabilities based on their own situation	0/49	
Place probabilities in context of other events	0/49	The risk of having an abortion was often described in relative terms and the “risk” was not defined (eg “far less than the risk of carrying a pregnancy and giving birth”)
Use both positive and negative frames	0/49	Frequently gave success or failure rates, but rarely provided both.
Describe the procedures and outcomes to help patients imagine what it is like to experience their physical, emotional and social effects	17/49	All included some description of the methods' physical effects, but often omitted the emotional and/or social effects of one or both methods.
Ask patients to consider what positive and negative features matter most	29/49	Majority provided implicit values clarification methods (eg table, list of pros and cons, list of reasons why women choose one vs the other) and omitted explicit values clarification methods.
Suggest ways for patients to share what matters most with others	2/49	
Provide steps to make a decision	2/49	
Suggest ways to talk about the decision with a health professional	3/49	
Include tools to discuss options with others	7/49	Infrequently provided question lists, which sometimes had limitations (eg only included questions for surgical abortion or listed generic and not applicable questions, such as “will this surgery be laparoscopy or open surgery?”). Women were rarely encouraged to write down their own questions.
*Development*		
Able to compare positive and negative features of options	28/49	The type, amount and organization of information given for each method was often inconsistent. Information was commonly presented in blocks of text under subcategories (eg side‐effects) or as answers to frequently asked questions, yet the content was often presented in different orders and with varying levels of detail. When information was presented in a table (n = 18), there was more consistency and equitable detail.
Show negative and positive features with equal detail	24/49	The majority described the positive or negative features inconsistently, with the negative features emphasized more often.
Include developers' credentials/qualifications	9/49	
Find out what users need to discuss options	0/49	
Has peer review by patient/professional experts not involved in development and field testing	0/49	
Is field tested with users	0/49	
The field tests with users show the decision aid is acceptable	0/49	
The field tests with users show the decision aid is balanced for undecided patients	0/49	
The field tests with users show the decision aid is understood by those with limited reading skills	0/49	
Provide reference to evidence used	6/49	
Report steps to find, appraise, summarize evidence	0/49	
Report date of last update	49/49	
Report how often patient decision aid is updated	2/49	
Describe the quality of the scientific evidence	0/49	
Use evidence from studies of patients similar to those of target audience	6/49	
Report source of funding to develop and distribute the decision aid	0/49	
Report where authors or their affiliations stand to gain or lose by choices patients make after using the decision aid	0/49	
Is written at a level that can be understood by the majority of patients in the target group.	9/48	
Is written at a grade 8 equivalent level or less according to readability score	11/48	The average US grade level required to understand the material was 9.6 (range: 5.1‐12) and the reading ease was 51.3 (range: 31.7‐65.1).
Provide ways to help patients understand information other than reading	33/49	The majority indicated that in‐person counselling would be available.
Provide a step‐by‐step way to move through the web pages	34/46	
Allow patients to search for key words	12/46	
Provide feedback on PHI that is entered into the patient decision aid	0/0	
Provide security for PHI entered into the decision aid	0/0	
Make it easy for patients to return to the decision aid after linking to other web pages	20/46	
Permit printing as a single document	14/46	
Use stories that represent a range of positive and negative experiences	0/4	
Report if there was a financial or other reason why patients decide to share their story	0/4	
State in an accessible document that the patient gave informed consent to use their stories	0/4	
*Effectiveness*		
Recognize a decision needs to be made	0/49	No evidence of evaluations was found.
Know options and their features	0/49	
Understand that values affect decision	0/49	
Be clear about option features that matter most	0/49	
Discuss values with their practitioner	0/49	
Become involved in preferred ways	0/49	
Improve the match between the chosen option and the features that matter most to the informed patient	0/49	

## 4 DISCUSSION

This systematic review and environmental scan found that very limited research has examined the impact of early abortion method decision aids, and although many are highly accessible, their quality scores are suboptimal. The low scores can be attributed, in part, to many decision aids describing method features inconsistently and with unequal detail, and presenting information in a disorganized fashion, potentially undermining perceptions of balance among users^42^ and impeding values‐consistent decision making. This is likely exacerbated among women with low literacy given that the majority of decision aids did not meet readability standards.^38^

The poor quality of existing early abortion methods decision aids not only represents a lost opportunity for supporting women's decision making, but may also affect their care experiences. For example, most tools did not describe the emotional and/or social effects of one or both methods transparently, so women who are concerned with these attributes may develop preferences for a less appropriate method. Because some women report choosing their method before approaching the health system,^9^ health professionals who offer abortion services, counselling and/or referral may need to be prepared to spend more time addressing such misperceptions and offering evidence‐based counselling, particularly among women with low literacy. This exchange may be particularly challenging in states that mandate the delivery of information during abortion counselling that is both inaccurate and not informed by patient preferences,^14^, ^43^ which some providers believe interferes with patient‐provider trust and rapport.^44^ This exchange may also lead women to opt for the alternative method, potentially inconveniencing both the patient and clinic with additional costs and logistical burdens that could have been avoided. By partnering with women and health professionals (ie end‐users) to understand their decision support priorities (eg optimal information content, presentation and delivery), there is significant opportunity to develop a more quality, relevant and sustainable^45^, ^46^, ^47^ early abortion method decision aid to promote patient‐centred and efficient abortion care.^48^

This study has implications for the current debate on decision aid certification.^49^, ^50^, ^51^ Our finding that, for this topic, decision aids produced by entities without specialist expertise in decision aid design predominate suggests that a certification approach that encompasses only tools produced by specialist developers may miss most of the decision aids accessible to patients via the Internet or other channels. Although reviewing all accessible decision aids for certification is clearly unrealistic, we recommend attention be paid to strategies for supporting patients to seek and recognize high‐quality, certified decision aids, and to understand the reasons for their superiority. Furthermore, our finding that most of the decision aids met limited criteria on the 64‐item IPDAS checklist suggests discordance between the priorities of subject matter experts and non‐specialist developers. A certification process that adopts more parsimonious quality criteria, such as the IPDAS minimum standards,^52^ may be the most suitable for ensuring decision aids achieve acceptable quality but can still be tailored to reflect real‐world needs.^53^ Simultaneously, efforts to facilitate non‐specialist developers' understanding of best practice standards for decision development and evaluation, and to encourage submission of decision aids for review and certification are warranted.

There are four main limitations of this study. First, because our focus was on understanding the early abortion method decision aid landscape for women in the United States, our systematic review excluded studies published before 2000 and thus may have omitted studies conducted earlier in countries with a longer history of performing medical abortion. Second, because we elected not to contact decision aid developers to solicit further information about development and evaluation processes, we may have underestimated the quality of some decision aids on these IPDAS domains. Third, because it was the most comprehensive, we adopted the 64‐item IPDAS checklist for decision aid appraisal. To our knowledge, this checklist has not previously been used to appraise decision aids developed by non‐specialists and thus may be less well suited to accurately capture the strengths and weaknesses of these tools. Lastly, by excluding studies evaluating decision coaching without an accompanying decision aid, we were unable to investigate the impact of different approaches to decision support in this context.

These limitations are, however, balanced by several strengths. First, we adopted an environmental scan methodology in addition to the more traditional systematic review, which proved essential for reliably understanding the current decision aid landscape in this area. We highly recommend that an environmental scan methodology be adopted more widely in attempts to understand the resources or interventions available to patients. Second, unlike a prior study that adopted an environmental scan,^54^ our methodology purposefully sought to identify tools that met the Cochrane definition of a decision aid, whether developed by specialists or non‐specialists. This prior study started with a baseline understanding of a decision aid and included only those tools that had a certain focus (ie prenatal testing), which likely missed some of the decision aids created by non‐specialists. Third, we used reliable screening and appraisal tools for the systematic review and environmental scan, evidenced by the adequate/high agreement between independent reviewers.

## 5 CONCLUSIONS

This systematic review and environmental scan demonstrate that research examining the impact of decision aids on early abortion methods is lacking, despite their potential to address key barriers to women's decision making process. Although many decision aids on this topic have been developed and made accessible in the United States, they are mostly poor quality and possibly undermining, rather than enhancing, quality decision making by women. Adapting an existing decision aid or developing a new decision aid on early abortion methods in partnership with end‐users and consistent with best practice decision aid standards is recommended.

## CONFLICT OF INTEREST

All authors have completed the ICMJE form for disclosure of potential conflict of interests. Kyla Donnelly has nothing to disclose. Glyn Elwyn reports personal fees from Emmi Solutions, LLC, personal fees from Washington State Health Department, personal fees from Oxford University Press, personal fees from Radcliff Press and grants from National Quality Forum outside the submitted work. Professor Elwyn reports ownership in the copyright of the CollaboRATE measure of shared decision making, the Observe OPTION measure of shared decision making, and several patient decision aids but has not received any personal fees connected to this copyright ownership. Rachel Thompson reports ownership in the copyright of several patient decision aids but has not received any personal fees connected to this copyright ownership. Dr. Thompson was an editor of the text, “Shared Decision Making in Health Care” and may receive royalties connected to this role in the future.

## Notes

1 Decision aids that included other pregnancy options (eg adoption and/or continuing the pregnancy) were included so long as they also compared medical and surgical early abortion methods.

2 There were two exceptions for Internet‐based decision aids: those that comprised only one tab and did not provide external links were coded as having met Items 44 and 48, respectively.
