# Supplementary material for: Quantity over quality—Findings from a systematic review and environmental scan of patient decision aids on early abortion methods
Source: Health Expect. 2017 Sep 7;21(1):316–26. doi: 10.1111/hex.12617 (PMC5750699; doi:10.1111/hex.12617)
Supplement: Supplementary file 1 [file HEX-21-316-s001.docx]

**IPDAS criteria modifications**

|  | **Original** | **Adapted (if relevant)** |
| --- | --- | --- |
| **I. Content: Does the patient decision aid…** | | |
| ***Provide information about options in sufficient detail for decision-making?*** | | |
| 1 | Describe the health condition | Describe the health condition (i.e., abortion; e.g., describes that abortion/termination is performed to end a pregnancy) |
| 2 | List the options | List the options (i.e., includes at minimum both surgical and medical abortion in early pregnancy) |
| 3 | List the option of doing nothing | List the option of doing nothing  (if yes, what are the option(s): [ ] waiting [ ] adoption [ ] continue the pregnancy) |
| 4 | Describe the natural course without options | Describe the natural course without options (i.e., describes the process of continuing a pregnancy) |
| 5 | Describe procedures | Describe procedures |
| 6 | Describe positive features (benefits) | Describe positive features (benefits) (e.g., success rates) |
| 7 | Describe negative features of options (harms / side effects / disadvantages) | Describe negative features of options (harms / side effects / disadvantages) (e.g., pain, failure rates, symptoms) |
| 8 | Include chances of positive / negative outcomes |  |
| ***Present probabilities in an unbiased and understandable way?*** | | |
| 9 | Use event rates specifying the population and time period |  |
| 10 | Compare outcome probabilities using the same denominator, time period, scale |  |
| 11 | Describe uncertainty around probabilities | Describe uncertainty around probabilities (e.g., explicitly says something along the lines of ‘cannot predict what exactly will happen to you’ or includes language such as ‘the [side effect] may happen’ or ‘women experience abortion differently’) |
| 12 | Use visual diagrams |  |
| 13 | Use multiple methods to view probabilities (words, numbers, diagrams) | Use multiple methods to view probabilities (words, numbers, diagrams) (e.g., 0.1% (1 in 1000) of women will have very heavy bleeding) |
| 14 | Allows the patient to select a way of viewing probabilities (words, numbers, diagrams) |  |
| 15 | Allow patient to view probabilities based on their own situation (e.g. age) | Allow patient to view probabilities based on their own situation (e.g., gestation) |
| 16 | Place probabilities in context of other events |  |
| 17 | Use both positive and negative frames (e.g., showing both survival and death rates) | Use both positive and negative frames (e.g., showing both success and failure rates) |
| ***Include methods for clarifying and expressing patients’ values?*** | | |
| 18 | Describes the procedures and outcomes to help patients imagine what it is like to experience their physical, emotional, social effects | Describes the procedures and outcomes to help patients imagine what it is like to experience their physical, emotional, and social effects (i.e., must describe all effects for both methods—i.e., will not meet criteria if only describes physical aspects; emotional examples: anxious waiting for the abortion process to be complete, viewing the pregnancy, anxious in a medical setting; social examples: concealing the abortion, privacy, availability of support person, childcare and work responsibilities, etc.) |
| 19 | Asks patients to consider what positive and negative features matter most | Asks patients to consider what positive and negative features matter most (e.g., implicit methods such as a table, list of reasons some women prefer the method) |
| 20 | Suggests ways for patients to share what matters most with others | Suggests ways for patients to share what matters most with others (e.g., encourages patients to voice what matter most to them; prompts patients to print out the tool to discuss with their provider) |
| ***Include structured guidance in deliberation and communication?*** | | |
| 21 | Provide steps to make a decision |  |
| 22 | Suggest ways to talk about the decision with a health professional |  |
| 23 | Include tools (worksheets, question list) to discuss options with others |  |
| **II. Development process: Does the patient decision aid…** | | |
| ***Present information in a balanced manner?*** | | |
| 24 | Able to compare positive / negative features of options | Able to compare positive / negative features of options (e.g., table, lists or reasons to choose one versus the other) |
| 25 | Shows negative / positive features with equal detail (font, order, display of statistics) |  |
| ***Have a systematic development process?*** | | |
| 26 | Includes developers’ credentials / qualifications | Includes developers’ credentials / qualifications (i.e., name of developer can be a person or research collaborative. If a clinic website, must have a designated person with their credential and qualifications associated with information) |
| 27 | Finds out what users (patients, practitioners) need to discuss options | Finds out what users (patients, practitioners) need to discuss options (i.e., either directly, such as through focus group, survey, or provider consultation, or indirectly, through literature search) |
| 28 | Has peer review by patient / professional experts not involved in development and field testing |  |
| 29 | Is field tested with users (patients facing the decision; practitioners presenting options) |  |
| 30 | The field tests with users (patient, practitioners) show the decision aid is acceptable |  |
| 31 | The field tests with users (patient, practitioners) show the decision aid is balanced for undecided patients |  |
| 32 | The field tests with users (patient, practitioners) show the decision aid is understood by those with limited reading skills |  |
| ***Use up to date scientific evidence that is cited in a reference section or technical document*** | | |
| 33 | Provides reference to evidence used |  |
| 34 | Report steps to find, appraise, summarize evidence |  |
| 35 | Report date of last update |  |
| 36 | Report how often patient decision aid is updated |  |
| 37 | Describes the quality of the scientific evidence (including lack of evidence) |  |
| 38 | Uses evidence from studies of patients similar to those of target audience |  |
| ***Discloses conflicts of interest?*** | | |
| 39 | Report source of funding to develop and distribute the decision aid | Report source of funding to develop and distribute the decision aid (i.e., for clinic websites, must have explicit information about funding used to create information about early abortion) |
| 40 | Reports where authors or their affiliations stand to gain or lose by choices patients make after using the decision aid | Reports where authors or their affiliations stand to gain or lose by choices patients make after using the decision aid (i.e., for clinic websites, must have explicit information that they do not stand to gain or lose from patients decision about medical versus surgical abortion) |
| ***Use plain language?*** | | |
| 41 | Is written at a level that can be understood by the majority of patients in the target group |  |
| 42 | Is written at a grade 8 equivalent level or less according to readability score (SMOG or FRY) | Is written at a grade 8 equivalent level or less according to readability score (Flesch-Kincaid) |
| 43 | Provides ways to help patients understand information other than reading (audio, video, in-person discussion) |  |
| ***Meet additional criteria if Internet based*** | | |
| 44 | Provide a step-by-step way to move through the web pages |  |
| 45 | Allow patients to search for key words |  |
| 46 | Provide feedback on personal health information that is entered into the patient decision aid |  |
| 47 | Provides security for personal health information entered into the decision aid |  |
| 48 | Make it easy for patients to return to the decision aid after linking to other web pages | Make it easy for patients to return to the decision aid after linking to other web pages (e.g., the link opens a new webpage so you do not lose the current webpage) |
| 49 | Permit printing as a single document |  |
| ***Meet additional criteria if stories are used in the decision aid*** | | |
| 50 | Use stories that represent a range of positive and negative experiences | Use stories that represent a range of positive and negative experiences (i.e., stories must be about the choice between methods, not review of clinic’s services or decision to have an abortion in general) |
| 51 | Reports if there was a financial or other reason why patients decide to share their story |  |
| 52 | State in an accessible document that the patient gave informed consent to use their stories | State in an accessible document that the patient gave informed consent to use their stories (i.e., applies to anonymous quotes) |
| **III. Effectiveness: Does the decision aid ensure decision-making is informed and values based?** | | |
| ***Decision processes leading to decision quality. There is evidence that the patient decision aid helps patients to:*** | | |
| 53 | Recognize a decision needs to be made | Recognize a decision needs to be made (e.g., Preparation for Decision Making Scale) |
| 54 | Know options and their features | Know options and their features (e.g., feel informed, e.g., “informed subscale” of DCS) |
| 55 | Understand that values affect decision | Understand that values affect decision |
| 56 | Be clear about option features that matter most | Be clear about option features that matter most (e.g., “values clarity subscale” on DCS) |
| 57 | Discuss values with their practitioner | Discuss values with their practitioner (e.g., PICS, CollaboRATE) |
| 58 | Become involved in preferred ways | Become involved in preferred ways (e.g., CPS) |
| ***Decision quality. There is evidence that the patient decision aid:*** | | |
| 59 | Improves the match between the chosen option and the features that matter most to the informed patient |  |
