# Supplementary figures and images for: Quantity over quality—Findings from a systematic review and environmental scan of patient decision aids on early abortion methods
Source: Health Expect. 2017 Sep 7;21(1):316–26. doi: 10.1111/hex.12617 (PMC5750699; doi:10.1111/hex.12617)

**Search results**

**PubMed 1904**

2/18/15

**
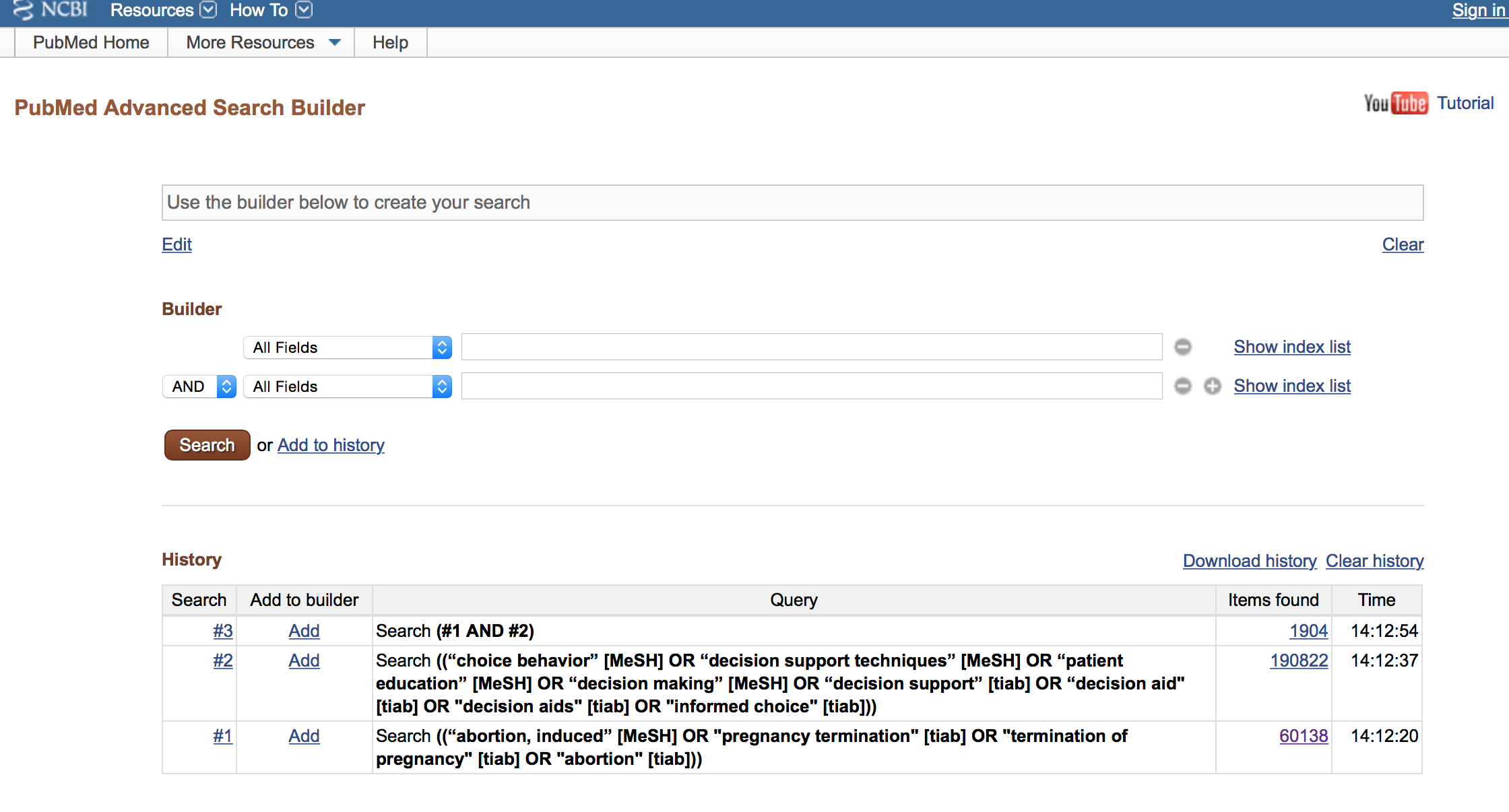
**

**Cochrane 112**

2/18/15

**
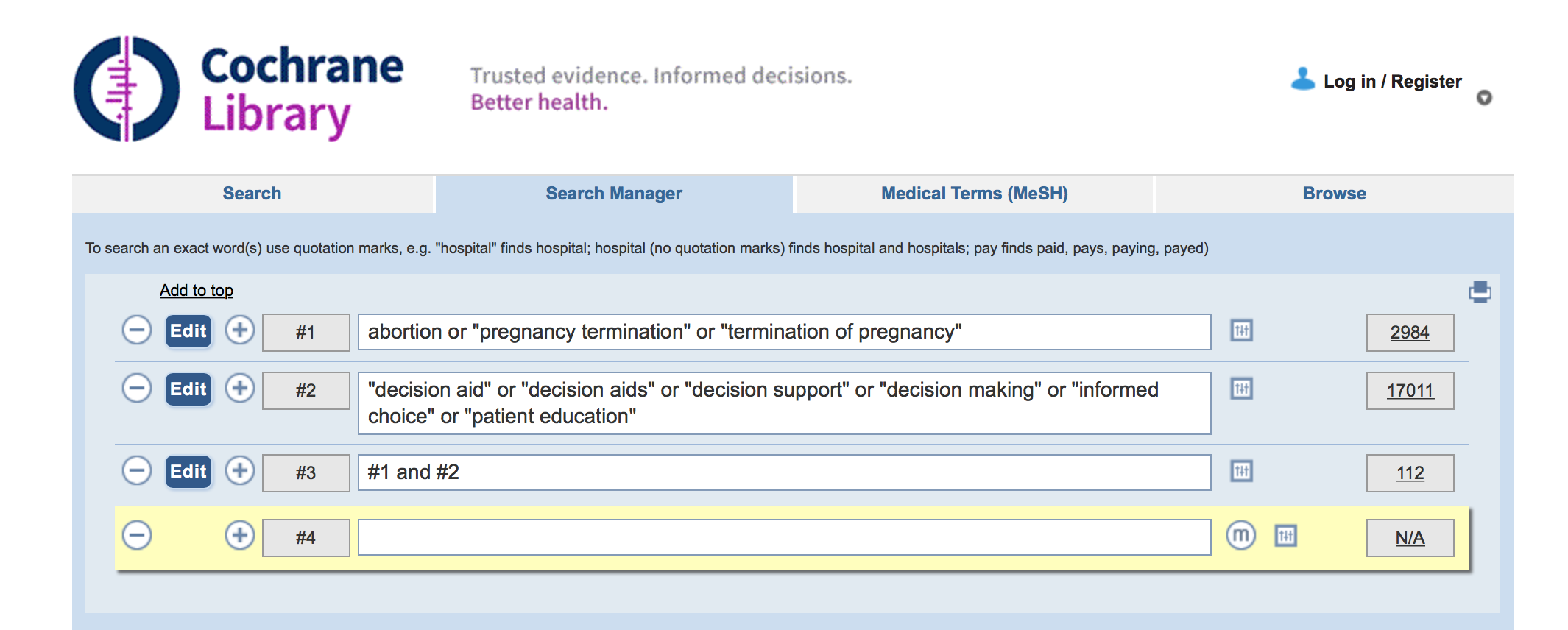
**

**Clinical Trials.gov 18**

2/18/15

**
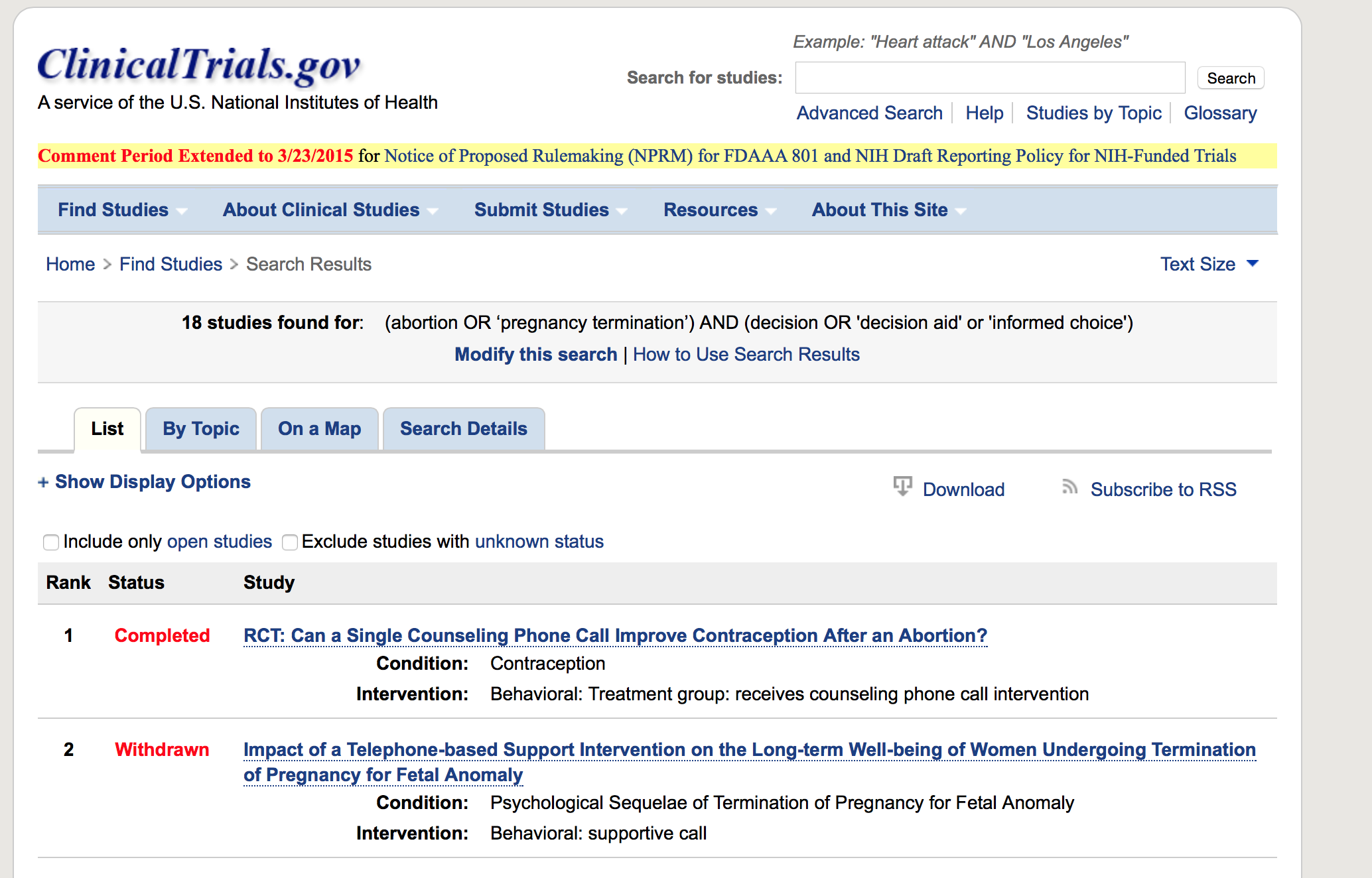
**

**PsycInfo 394**

2/18/15

**
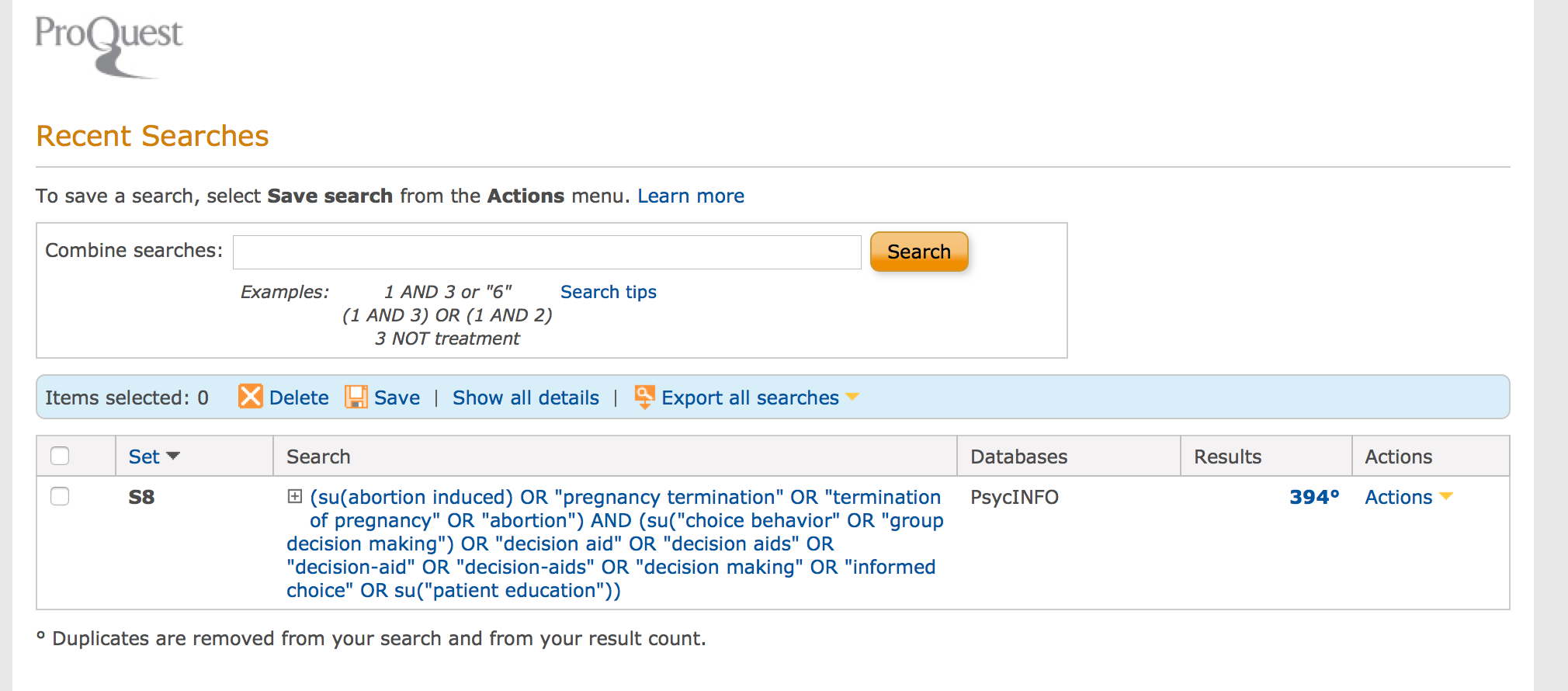
**

**Cinahl 292**

2/18/15

**
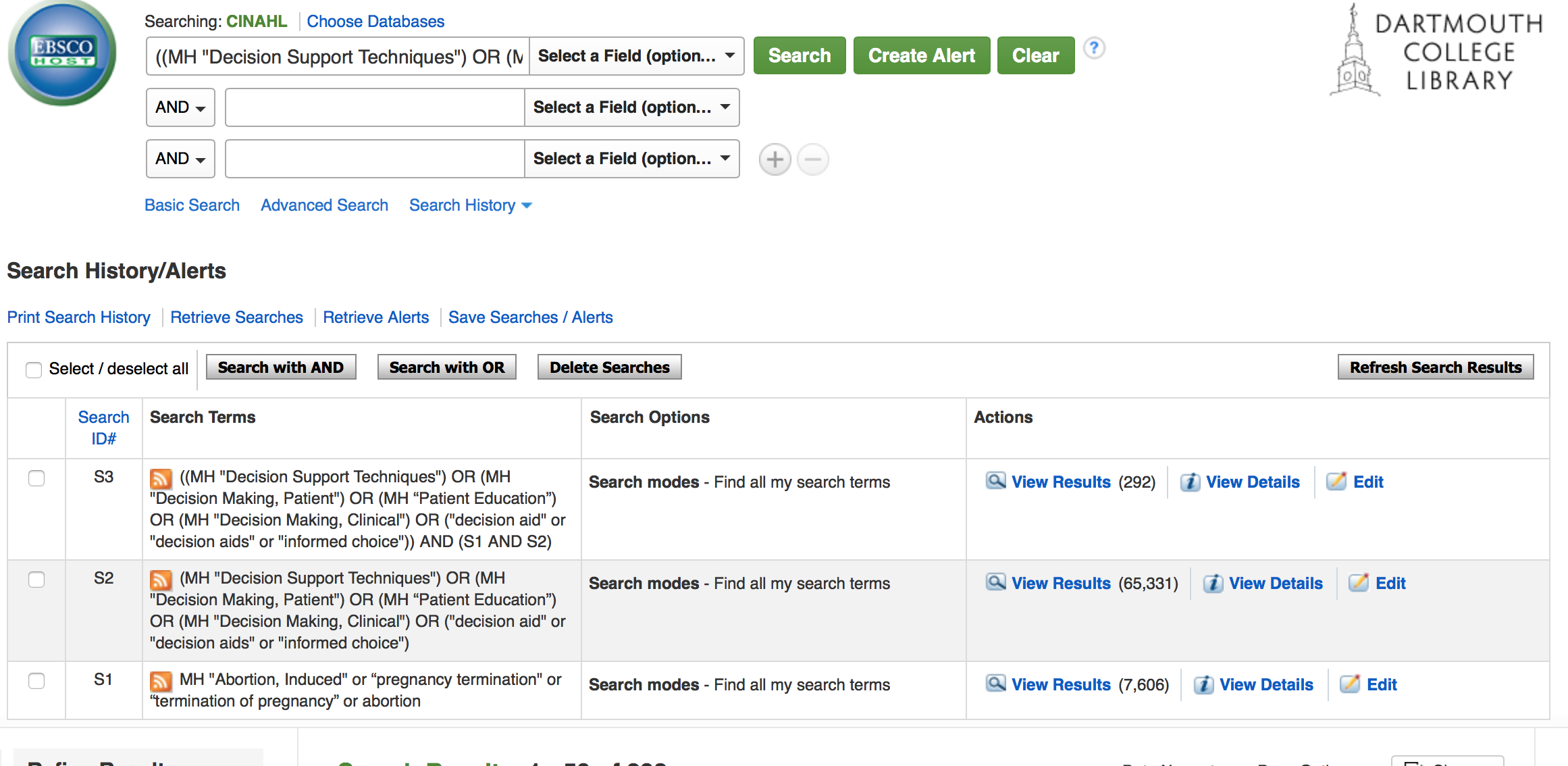
**

**Embase**

2/23/15


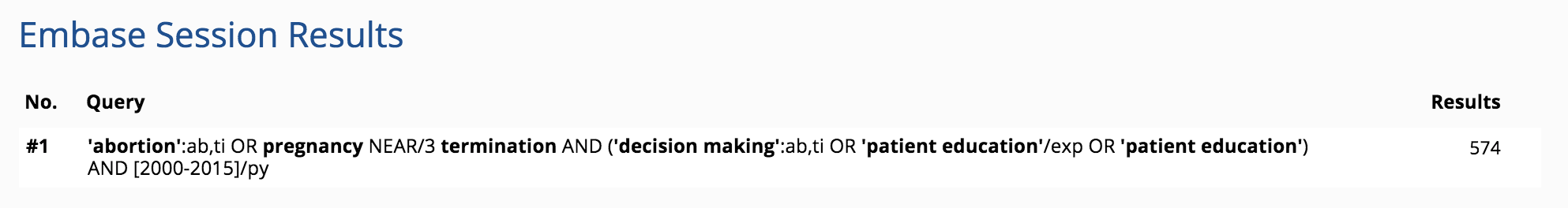

Supplement: Supplementary file 2 [file HEX-21-316-s002.docx]
